# Development of Specific Motor Skills through System Wall Bouldering Training: A Pilot Study

**DOI:** 10.1155/2024/5584962

**Published:** 2024-07-08

**Authors:** Nicolay Stien, Kaja Langer, Vidar Andersen, Gunn Helene Engelsrud, Elias Olsen, Atle Hole Saeterbakken

**Affiliations:** ^1^ Faculty of Education Arts and Sports Western Norway University of Applied Sciences, Sogndal, Norway; ^2^ Department of Human Sciences Institute of Sports Science Technical University Darmstadt, Darmstadt, Germany

## Abstract

This study evaluated the effects of a five-week period of practicing specific climbing movements using a system wall on motor skills and bouldering performance compared to self-regulated, conventional bouldering. Thirteen advanced female boulderers (age: 24.5 ± 3.6 years, height: 166.9 ± 3.4 cm, and body mass: 63.4 ± 8.0 kg) were divided into an experimental group (*n* = 7) and a control group (*n* = 6). Both groups continued their normal training routines during the intervention, but the experimental group dedicated 30 minutes of their climbing time twice per week to practicing specific motor skills on a system climbing wall. Before and after the intervention, the participants attempted two boulder problems on the same wall. The performance was registered as the number of attempts to complete the boulder problems and as the highest hold reached within four attempts. Video recordings of climbers' best attempts, capturing the highest hold reached from a perspective directly behind them, were analyzed by three independent experts. The analysis was conducted using a five-point scale across six categories of movement quality. Modest enhancements in certain motor skills and performance were evident in both groups, revealing no significant distinction between them. The results underscore the efficacy of incorporating system walls into the training routines of advanced female boulder climbers, but the absence of between-group differences highlights the significance of individual preferences when choosing between conventional and system wall bouldering.

## 1. Introduction

Recent years have witnessed an increased interest in climbing among professional and recreational circles. While the enhancement of sport-specific fitness in male climbers at higher performance levels has been extensively examined [1], there is a notable gap in research concerning recreational female climbers [2]. This group's engagement with resistance training is lower [3, 4], underscoring the need to explore climbing-specific activities that could improve their performance. The importance of strength is recognized [5–7], yet the critical role of motor skills, for instance, executing efficient movements and adjusting body positions swiftly [8, 9], should not be overlooked [10].

In modern commercial bouldering gyms, the volume of available problems provides climbers with a wide array of challenges. While this variety may encourage exploration of different climbing styles and movement types, it can also inadvertently steer climbers to seek routes that align with their preferred climbing variety (e.g., hold types and wall steepness). This natural inclination towards familiar types of climbing, coupled with the vast selection, may lead climbers to engage less frequently in the repetitive practice of specific problems in their self-regulated training. Such repetition is essential for errorless learning and motor skill refinement [11–13]. Although climbers probably revisit problems they have completed, either for enjoyment or as part of their training regimen, the tendency to continuously seek new challenges might limit the opportunities for focused skill development through repeated attempts at the same problem. Previous studies suggest that structured climbing training can enhance performance without necessarily increasing the frequency or training intensity [14, 15]. Leveraging the diversity of problems while also encouraging targeted repetition could address the gaps in current practice and promote more comprehensive skill development among climbers.

Recently, walls equipped with lights integrated in the holds and adjustable steepness have emerged as novel training tools in bouldering. Such walls are capable of displaying a multitude of community-created boulder problems with consensus-based difficulty rating which can be accessed by a phone application. This type of wall presents a potentially useful tool for standardizing performance measures, quantifying and tailoring training, and adapting the difficulty and style to all levels of climbers. Using technologically augmented walls for climbing training may hold potential, especially if their effectiveness in enhancing motor learning and skill development can be demonstrated. By incorporating automated, visual instruction in climbing training, it further becomes possible to optimize the allocation of human resources by reducing the hands-on time required by a trainer. Despite their potential benefits, such walls remain underexplored in research.

Acknowledging the gap in climbing literature on movement skill development and recreational, female climbers [2, 8], our pilot study evaluates the effects and feasibility of a five-week training period using a system wall on motor skills and bouldering performance compared to the same duration of self-regulated, conventional bouldering training. Given the theory that structured, repetitive practice can lead to significant improvements in climbing-related fitness and performance [11, 12, 14, 15], we hypothesized that the deliberate practice on prescribed boulder problems on a system wall would result in greater improvements in motor skills and bouldering performance compared to the self-regulated bouldering training.

## 2. Materials and Methods

### 2.1. Experimental Design

An experimental trial with two groups was employed to investigate the research question. Prior to the commencement of the intervention, all participants were assessed for bouldering performance and technical execution. The group assignment was determined by randomly drawing lots from an opaque container immediately following the completion of all pretesting procedures. The intervention lasted five weeks and consisted of two 30-minute sessions per week. The sessions included training on a system wall (Kilter, LLC, Boulder, Colorado, United States) attempting a selection of ten boulder problems that were designed to target specific movement skills (i.e., accuracy, balance, sequencing, technique, arm use, and movement initiation). The control group, meanwhile, continued with their usual bouldering and training regimens, with an instruction to abstain from using the system wall during the intervention period.

### 2.2. Participants

Given the absence of comparable studies, a comprehensive power analysis could not be performed. Nevertheless, diligent efforts were made to enlist a maximal number of eligible participants within the local context. This involved reaching out to all eligible climbers in the local climbing club and consistently visiting the climbing gym to establish contact with potential participants. The final study population comprised 13 advanced, female boulder climbers (Table 1) who fit the inclusion criteria of having to be active climbers, defined as conducting at least one weekly climbing session, but not having completed a boulder problem graded higher than IRCRA 20 (International Rock-Climbing Research Association; scale ranges from 1 to 32). They were also required to not have used system wall climbing as part of their training program before enrolling in the study. Moreover, they had to be free of injuries or illnesses that could limit their ability to participate in the training or exert maximal effort in the testing. The recruitment phase spanned from September 26 to October 7, 2022, with participants maintaining their involvement in the study until the conclusion of all training and data collection. The last posttest assessment took place on November 20, 2022. The study procedures conformed to the latest revision of the Helsinki declaration, followed Norwegian laws and regulations, and were processed by the Norwegian Center for Research Data (reference: 992074) and the local ethics committee (reference: 21/08477-3).

### 2.3. Procedure

Two boulder problems were set to assess performance and movement skills. The climbers were given four attempts on each boulder problem with up to one minute rest between attempts on the same problem and two minutes rest between boulder problems. Adapting the approach of a similar study from our lab [16], the attempts on the two system wall boulders were recorded using a stationary iPad (Apple Inc., Cupertino, California, US) positioned approximately four meters away from the center of the wall, ensuring a perpendicular line of sight to the wall. Of the maximum four attempts, the best attempt was stored (i.e., the attempt in which the highest hold was reached). Three independent experts in the field with several years of experience as climbing trainers for national and international levels were then engaged to provide feedback on the recordings using a simplified version of the climber's movement performance assessment tool developed and validated by Taylor and colleagues [10]. The score sheet included six items (accuracy (ACC), balance/fluidity (BAL), sequencing/exploration (SEQ), technique (TEC), arm position (ARM), and movement initiation (MOV); see Table 2 for more information about each item), each using a five-point scale ranging from 1 (poor or nonexistent component) to 5 (flawless demonstration of skilled performance). Five random videos were displayed twice to facilitate the calculation of intrarater reliability. The experts were blinded to the participant's assigned group and unaware of whether they were viewing a pre- or posttest recording. To further ensure anonymity, any visible faces were removed before the footage was presented. Moreover, the experts were externally recruited and had no prior knowledge about the study design or the participants.

Performance was registered as the number of attempts required to complete the boulder problem or as the number of holds reached on their best attempt if they could not complete the boulder problem. In accordance with competition rules, one point was awarded for each handhold successfully grasped and utilized to move upward on the climb, while half a point was given for touching the next handhold, but not being able to sustain it. In addition, the climbing duration (time from the second foot left the floor until both hands were touching the top hold) was collected from the footage by using time stamps in a commercial video player software (VLC Media Player 3.0, VideoLAN, Paris, France). Climbing duration was only analyzed for the eight participants that completed the boulder problems at both pre- and posttest (three in the control group and five in the experimental group).

### 2.4. Intervention

The participants in the experimental group replaced 30 minutes of their regular climbing and training time to performing the movement skill training twice per week for five weeks, with at least one full day of rest between the two weekly sessions. The experimental group conducted their initial session under supervision and was presented with a selection of ten boulder problems, systematically increasing in difficulty, which they were to engage with on the system wall. The boulder problems were designed to progressively increase in difficulty by incorporating elements such as smaller footholds, more unbalanced positions, longer moves, and intricate sequences, as these would be relevant for the expert rating items. We deliberately minimized increases in difficulty that solely relied on reducing hold size, ensuring that technical skills remained more crucial than sheer strength. Subsequent sessions predominantly operated without direct supervision.

To guarantee the participants' compliance with the training protocol and to oversee the progression of their training, the researchers attended the fifth and tenth sessions. Our continuous presence was consistently upheld throughout the intervention period to address any queries or challenges participants encountered during the training. The participants were instructed to work on four problems in each session, beginning with the easiest four, and to spend no more than five minutes working on each problem before progressing to the next. If they completed a problem with relative ease, they could omit the problem from the following session and replace it with a more challenging one, while the remaining three from the preceding session were still included. Hence, each session lasted around 30 minutes including rest periods. For the remainder of their time in the gym, the participants climbed and trained as usual on conventional bouldering walls. The control group continued their regular routines throughout the intervention period.

### 2.5. Statistical Analyses

The analyses were performed using the commercial statistical software SPSS (IBM Corp. Released 2020. IBM SPSS Statistics for Windows, Version 27.0. Armonk, NY: IBM Corp). In addition to a Shapiro–Wilk test revealing primarily nonparametric distributions, the ordinal nature of much of the data along with the low number of participants, we opted for nonparametric statistical tests. Thus, between-group differences were assessed using Mann–Whitney *U*-tests, whereas within-group differences from pre- to posttest were addressed with Wilcoxon signed-rank tests. Intrarater reliability and interrater reliability were assessed with the intraclass correlation (ICC) of the absolute agreement, which considers both the degree of correlation and the agreement among raters, as well as between ratings, thereby evaluating if the ratings are correlated and also if they are identical in absolute terms. For the interrater reliability, all items were compared across the three raters. For intrarater reliability, ratings of the six items were compared across two ratings of the same video by each rater, and the average ICC for each reviewer was calculated. The choice to average all items in the reliability analyses was made due to the potential interrelated nature of different items (e.g., arm position and movement initiation) and to provide a more substantial value describing the reliability of each rater. Values within the range of 0.21–0.40 indicated fair agreement, 0.41–0.60 suggested moderate agreement, 0.61–0.80 was indicative of substantial agreement, and 0.81–0.99 signified near-perfect agreement [17]. Statistical significance was accepted at *p* < 0.05. Training compliance, number of attempts, and climbing time are displayed using median and range, while the remaining results are presented as means with standard deviations and effect sizes (ESs) for the changes. The ESs were calculated as the Z-score divided by the square root of the number of observations (*n*) and interpreted as trivial (<0.10), small (0.10–0.30), medium (>0.30–0.50), and large (>0.50) [18].

## 3. Results

### 3.1. Baseline and Training

The two groups were not different at pretest in any of the collected variables (*p*=0.093 − 0.779). The study participants self-identified predominantly as boulderers and reported dedicating an average of 64 ± 21% of their climbing time to the bouldering discipline. The training group reached a median compliance rate of 10 bouldering sessions (range: 9-10), whereas the control group completed a median of 9 (range: 8–11) bouldering sessions during the same five weeks. The experimental group's sessions lasted 67.5 ± 18.4 minutes, plus 30 minutes of prescribed system wall training, while the control group's sessions averaged 101.0 ± 16.7 minutes. When including the 30 minutes of system wall training for the experimental group (average: 97.5 ± 18.4 minutes), the training times were not different between the groups (ES = 0.05, *p*=0.751).

### 3.2. Expert Ratings

**Figure 1:**
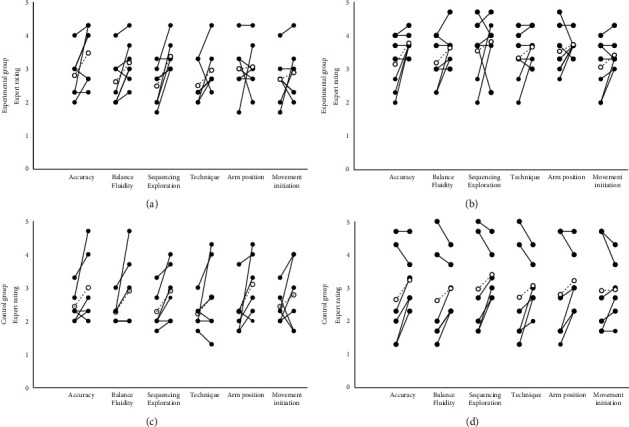
Overview of the individual expert ratings received on the 1–5 scale. (a) and (b) illustrate changes for the experimental group on boulder problems A and B, respectively, while the changes for the control group on problems A and B are shown in (c) and (d), respectively. The average change is indicated by white markers and asterisks indicate significant changes from pre- to posttest.

On boulder A, the experimental group improved their scores for BAL (0.57 ± 0.56, ES = 0.29, *p*=0.047) and SEQ (0.91 ± 0.66, ES = 0.43 *p*=0.026) at posttest, whereas the control group only improved at SEQ (0.61 ± 0.49, ES = 0.19, *p*=0.042; Figure 1). Neither group achieved a change in scores on boulder B (*p*=0.068–0.783, ES = 0.01–0.06). None of the expert ratings were different between the groups at pre- (*p*=0.126–0.569, ES = 0.09–0.23) or posttest (*p*=0.113–886, ES = 0.05–0.23), nor were any of the change scores different (*p*=0.097–0.664, ES = 0.01–0.26). The groupwise changes for each item across the two boulder problems are presented in Table 3. See Supplementary Tables 1 and 2 for all individual ratings on each boulder problem separately. For the individual data, improvements in expert ratings were generally more pronounced for participants receiving low scores (<2) at pretest, whereas high pretest scores were generally associated with negligible improvements, and in some cases with declines.

### 3.3. Intrarater Reliability

For the intrarater reliability across all items, the ICC demonstrated near perfect agreement for two of the raters (ICC = 0.894 ± 0.062 and ICC = 0.895 ± 0.060), whereas one exhibited substantial agreement (ICC = 0.807 ± 0.181). Note that all analyses were conducted both with and without the involvement of the third rater (the rater with marginally lower reliability score), and the resulting differences in the outcomes were negligible and did not affect any of the results.

### 3.4. Interrater Reliability

The interrater reliability across the six variables ranged from 0.747 to 0.850 with an average of 0.795 ± 0.039. The raters reached substantial agreement for ACC (ICC = 0.799), SEQ (ICC = 0.757), ARM (ICC = 0.794), and MOV (ICC = 0.747) and near perfect agreement for BAL (ICC = 0.850) and TEC (ICC = 0.820). Sensitivity analyses with exclusion of the rater with weaker reliability resulted in a slight reduction in the mean ICC (0.786 ± 0.041), further supporting the decision to include all three raters in the analyses.

### 3.5. Bouldering Performance

For bouldering performance, five of the seven participants in the experimental group managed to climb boulder problem A in the pretest, whereas all of them completed it in the posttest. Five of the six participants in the control group completed both boulder problems during the pretest and all were able to do it at posttesting. For boulder problem B, all the participants in the experimental group managed the boulder problem within four attempts at both pre- and posttest, whereas the number of tries increased from four to six (all participants) from pre- to posttest in the control group.

### 3.6. Attempts

There were no changes in the number of attempts to complete the two boulder problems from pre- to posttest in either group (ES = 0.37-0.74, *p*=0.317–1.000), and no significant differences were observed between groups at any testing point (ES = 0.05–0.22, *p*=0.255–0.904). Initially, the experimental group's median number of attempts for boulder problem A was 1 (range: 1–3), while the control group required a median of 2 (range: 1–3). By the posttest, the experimental group required a median of 1 (range: 1–3), with the control group slightly improving to a median of 1.5 (range: 1–3). For boulder problem B, both groups started with a median of 1 attempt; the experimental group range was 1-2, and the control group range was 1–4. At the posttest, the experimental group remained at a median of 1 (range: 1–3), while the control group needed a median of 1.5 attempts (range: 1–3).

### 3.7. Climbing Duration

Among participants who completed both boulder problems at pre- and posttest (three in the control group and five in the experimental group), the experimental group decreased their median climbing time for boulder problem A from 46 seconds (range: 31–63) to 33 seconds (range: 29–40; ES = 0.46, *p*=0.049). There was no significant reduction in time for boulder problem B (ES = 0.19, *p*=0.179) in this group. In contrast, the control group's climbing times for both boulder problems A and B remained consistent (ES = 0.03, *p*=0.894 and ES = 0.09, *p*=0.198, respectively). Individual data analysis revealed more pronounced reductions in climbing time in the experimental group, with a median decrease of 9.5 seconds (range: 1–37 seconds), compared to a minor median reduction of 2 seconds (range: 1–8 seconds) in the control group. Additionally, climbing times increased for two control group participants by 14 and 3 seconds on boulder problems A and B, respectively, whereas increases in the experimental group were confined to boulder problem B, with two participants experiencing increases of 1 and 5 seconds.

## 4. Discussion

This study sought to compare the effects of climbing technique practice using a system wall and self-regulated, conventional bouldering training on motor skills among advanced, female boulderers. While our findings offer preliminary insights, they also highlight the necessity for studies with larger participant pools to enhance the robustness and generalizability of the results. Both groups exhibited improvements posttraining, with significant enhancements in balance and fluidity (BAL) noted only in the experimental group. Importantly, the low number of participants and the marked individual differences must be considered when interpreting the results.

The BAL metric gauges a climber's adeptness at maintaining a seamless flow of movement while avoiding destabilization [10]. The system wall training focused participants on specific movement patterns, enhancing cognitive engagement to refine motor skills through movement analysis and targeted improvement. Conversely, the control group's BAL ratings did not improve, possibly due to the variable nature of self-regulated training, which may lack the structured practice needed to sharpen specific skills [11, 12]. Importantly, this argument warrants further investigation into how recreational climbers structure their self-regulated training. It should also be noted that these findings are influenced by two of the participants in the control group receiving lower ratings for BAL at posttest, whereas only one participant in the experimental group decreased in this metric. Interestingly, all three participants who decreased in the BAL rating initially received high scores at pretest (4 and 5), suggesting a potential ceiling effect where higher initial scores limit observable improvements. Future research should remain cautious of ceiling effects in performance measurements, especially when employing scales with upper limits. Such limitations can skew results, particularly when initial scores are high, as they limit the scope for detecting improvements. This phenomenon was evident in our pilot study and serves as a critical consideration for the design and interpretation of future experiments. Finally, training context differences—system wall versus conventional walls used by the experimental and control groups, respectively—could suggest that environmental factors like wall type and hold shapes might affect skill transferability, despite not practicing the exact boulder problems tested [19, 20].

One of the primary limitations of the current study is its low statistical power, a consequence of the small sample size. Future investigations should aim to include more participants to better detect small, but meaningful effects that could be obscured in smaller samples. However, analyzing individual performance reveals nuances not captured in broader analyses. Participants in the experimental group demonstrated improvements in ACC, BAL, and SEQ for both boulder problems. These improvements align with the group-level findings of enhanced balance and fluidity, as well as sequencing and exploration. In contrast, the control group displayed varied responses, with some participants improving and others showing no change or slight declines. Notably, one control group participant's significant improvement influenced the average change in boulder problem A, likely affecting the overall between-group comparison. These findings indicate that system wall training can enhance technical skills and bouldering performance for advanced female climbers, though variable results and lack of significant group differences underscore the importance of tailoring training to individual preferences and abilities.

Two control group participants scored high (>4) for all rated items on boulder problem B initially but showed notable declines at posttest, significantly influencing both the group's overall change and within-group variation. These outcomes could be interpreted to suggest that conventional, self-regulated training may be less effective for climbers already proficient in motor skills, while structured approaches like system wall training could offer greater benefits by focusing more on specific motor skills or deficiencies in their technique. Variability in training responses may also be influenced by individual differences such as climbing experience, learning preferences, and engagement with training. The substantial variability in individual responses to the interventions is an important takeaway from our findings. This variability suggests that future research should tailor analyses to account for individual differences, which may reveal more nuanced understandings of the factors influencing performance gains, especially among advanced, female boulderers. Additionally, filming only two boulder problems, despite providing more data than much existing literature, cannot fully represent all climbing-specific motor skills. Incorporating a broader range of movements could enrich our understanding of the system wall's impact as a training tool. Given these individual trends and the study's limited statistical power, cautious interpretation is needed, but these insights lay groundwork for further research with larger samples.

Through the prescription of ten boulder problems featuring a gradual escalation in difficulty, the system wall training method may have facilitated errorless learning. The errorless learning approach to bodily learning involves initiating the practice of a new skill at a lower level of complexity [13], thereby enabling participants to engage with minimal effort while implicitly honing technique and form, which can subsequently be applied to overcoming more demanding challenges. The findings further correspond with the concept of resonance, wherein the emergent motor actions resonate with the environmental demands, optimizing the energy expenditure through harmonious coordination [21]. In contrast, the self-regulated conventional bouldering likely involved a higher frequency of participants attempting boulder problems close to their skill limits, possibly resulting in a greater occurrence of errors in their movements and solutions. This heightened error frequency might have extended the time required for this group to identify effective attractors, adapt individualized solutions, and refine movements perceived as effective [22]. Again, further research into the habits of recreational climbers is warranted.

All participants completed both boulder problems within four attempts after the intervention, showing slight improvements for the control group in both problems, and for the experimental group in only boulder problem A. This change was accompanied by significant SEQ enhancements from pre- to posttest in both groups. Of note, the ES for experimental group (ES = 1.54) was approximately twice as large as for the control group (ES = 0.77), indicating that a higher statistical power likely could have detected between-groups differences. The SEQ metric, assessing the ability to determine optimal movement sequences, likely benefited from participants' familiarity with the problems, revisited five weeks later. While this recollection might have eased task completion, minor differences in training intensity and duration between the groups also potentially influenced the results.

The findings regarding minor differences between groups are comparable to a similar study on motor skill learning conducted in our lab which suggested that, albeit not superior to conventional training approaches, technologically augmented feedback may present a viable alternative which reduces the manual labor required for instructions [16]. Importantly, the completion of both boulder problems by all participants highlights a limitation in using such tasks for continuous performance assessment, since a ceiling effect is reached. When climbers are able to successfully complete the problems, assessing further improvements becomes impossible, as the task no longer differentiates between varying levels of performance. To address this, future studies should explore the use of higher numbers of boulder problems or climbs including more moves, as this could allow for a more nuanced perspective of the performance.

This study provides new insights on system wall training versus conventional bouldering but has some limitations. It focused solely on advanced female boulder climbers, limiting generalizability to other demographics like male climbers or those at different skill levels. The small sample size and the study's location in a small locale also restricted participant diversity. Despite these challenges, the results offer valuable insights and a basis for future studies. Additionally, the decision to include a third rater with weak reliability could be viewed as a limitation. Importantly, sensitivity analyses showed minimal impact from this decision on the outcomes. Potential group cross-contamination was mitigated by separate training facilities, although completely controlling this in such a social sport is challenging without geographical separation beyond what was feasible for this study. The five-week intervention period, while brief, did yield some significant skill improvements, suggesting that longer durations and more participants could provide further insights into the sustainability and variability of these enhancements. Future studies could explore longer interventions and more participants to better understand these effects.

Traditional studies on sports performance enhancement typically favor long-term training focused on physical variables like strength and endurance [23–26]. However, linking these improvements to real sport performance remains a challenge. This study took a different approach by adopting a shorter training period and focusing on specific climbs to evaluate effectiveness. This shift towards sport-specific assessments provides insights into climbers' skill proficiency, underlining the potential benefits of short-term, targeted training interventions for practitioners seeking quick performance gains. More importantly, our findings display the feasibility for future studies using similar designs, but with more statistical power and longer intervention durations.

## 5. Conclusions

The experimental group's system wall training led to enhancements in balance and fluidity, benefits not seen in the control group. Notably, no significant between-groups differences were detected. The experiences and findings from our research should be taken into account when designing future studies on the subject.

## Figures and Tables

**Table 1:** Study sample characteristics by groups and combined presented as mean ± standard deviation.

	Experimental group (*n* = 7)	Control group (*n* = 6)	Total (*n* = 13)
Age (years)	24.0 ± 3.3	24.3 ± 4.2	24.2 ± 3.6
Height (cm)	166.1 ± 4.1	167.8 ± 2.5	166.9 ± 3.4
Body mass (kg)	60.9 ± 9.8	66.2 ± 4.6	63.4 ± 8.0
Experience (years)	2.9 ± 2.5	2.1 ± 1.6	2.5 ± 2.1
Weekly sessions	2.6 ± 0.6	2.5 ± 1.2	2.6 ± 0.9
Best climb (IRCRA)	16.9 ± 1.9	16.9 ± 2.0	16.9 ± 1.8

IRCRA = International Rock-Climbing Research Association. Climbing grades are converted into a 1–32 numerical scale.

**Table 2:** Descriptions of the lowest and highest scores for the five-point simplified movement assessment tool adopted from Taylor et al. [10].

Item	Abbreviation	Description of lowest score (1)	Description of highest score (5)
Accuracy	ACC	Messy climbing and must adjust every hand and foot move	Hands and feet are placed precisely and accurately every time
Balance/fluidity	BAL	Always off balance and moves twitchy and uncontrolled	Perfect balance and fluidity in all movements
Sequencing/exploration	SEQ	Performs sequences inappropriately with a frequent and extended exploration of possible holds	Does all sequences in an appropriate and effective manner. Finds the right sequence right away with purposeful moves
Technique	TEC	Ineffective and unvaried solutions	Demonstrates a broad repertoire of skills that are used to solve the boulder appropriately
Arm position	ARM	Always climbs with bent arms	Climbs with straight arms when appropriate
Movement initiation	MOV	All movements are initiated with the arms	Uses momentum and lower body to generate momentum where applicable

**Table 3:** Mean and median changes in the expert ratings across the two boulder problems.

Group	Accuracy	Balance/fluidity	Sequencing/exploration	Technique	Arm position	Movement initiation
*Experimental*						
Mean change	0.7	0.6	0.9	0.4	0.5	0.2
SD	0.3	0.6	0.7	0.7	1.0	0.8
Median change	0.7	0.3	1.3	0.3	0.0	0.0
IQR	0.8	0.7	1.0	0.6	0.5	1.0

*Control*						
Mean change	0.6	0.6	0.6	0.5	0.8	0.3
SD	0.8	0.9	0.5	1.0	1.0	1.0
Median change	0.5	0.3	0.5	1.7	0.7	0.5
IQR	0.6	0.7	0.6	1.1	0.5	1.4

Mean change is displayed with standard deviations (SDs) and the median change is reported with interquartile ranges (IQRs).

## Data Availability

All data are available from the corresponding author upon reasonable request.

## Supplementary Materials

Supplementary Materials Supplementary Table 1: mean expert ratings on boulder problem A for the experimental (1) and control (2) groups. Supplementary Table 2: mean expert ratings on boulder problem B for the experimental (1) and control (2) groups.
