# Supplementary material for: Development of Specific Motor Skills through System Wall Bouldering Training: A Pilot Study
Source: Transl Sports Med. 2024 Jul 8;2024:5584962. doi: 10.1155/2024/5584962 (PMC11250695; doi:10.1155/2024/5584962)
Supplement: Supplementary Materials — Supplementary Table 1: mean expert ratings on boulder problem A for the experimental (1) and control (2) groups. Supplementary Table 2: mean expert ratings on boulder problem B for the experimental (1) and control (2) groups. [file 5584962.f1.zip › Supplementary table 1 (3).docx]

**Supplementary table 1:** Mean expert ratings on Boulder problem A for the experimental (1) and control (2) groups.

|  |  | **Accuracy** | | **Balance/fluidity** | | **Sequencing/exploration** | | **Technique** | | **Arm position** | | **Movement initiation** | |
| --- | --- | --- | --- | --- | --- | --- | --- | --- | --- | --- | --- | --- | --- |
| **Nr.** | **Group** | **Pre** | **Post** | **Pre** | **Post** | **Pre** | **Post** | **Pre** | **Post** | **Pre** | **Post** | **Pre** | **Post** |
| **1** | 1 | 2.0 | 2.7 | 2.0 | 3.0 | 1.7 | 3.0 | 2.0 | 2.7 | 3.0 | 2.7 | 2.7 | 2.3 |
| **2** | 1 | 3.0 | 4.0 | 3.0 | 3.3 | 2.7 | 3.0 | 3.3 | 2.3 | 3.3 | 3.0 | 2.7 | 2.3 |
| **3** | 1 | 2.3 | 4.3 | 2.3 | 3.7 | 2.0 | 3.7 | 2.0 | 3.3 | 1.7 | 3.7 | 1.7 | 3.3 |
| **4** | 1 | 3.0 | 2.7 | 3.0 | 2.7 | 3.3 | 3.3 | 2.3 | 2.7 | 2.7 | 2.7 | 3.0 | 3.0 |
| **5** | 1 | 4.0 | 4.3 | 4.0 | 4.3 | 3.0 | 4.3 | 3.3 | 4.3 | 4.3 | 4.3 | 4.0 | 4.3 |
| **6** | 1 | 2.3 | 2.3 | 2.0 | 2.3 | 2.7 | 3.0 | 2.3 | 2.7 | 3.3 | 2.0 | 2.7 | 2.0 |
| **7** | 1 | 3.0 | 4.0 | 2.0 | 3.0 | 2.0 | 3.3 | 2.3 | 2.7 | 2.7 | 3.0 | 2.0 | 3.0 |
| **8** | 2 | 3.3 | 4.0 | 3.0 | 3.7 | 3.3 | 3.7 | 3.0 | 4.0 | 3.7 | 4.0 | 3.3 | 4.0 |
| **9** | 2 | 2.3 | 2.3 | 2.0 | 2.0 | 1.7 | 2.0 | 2.0 | 2.0 | 2.3 | 3.3 | 2.0 | 3.0 |
| **10** | 2 | 2.7 | 4.7 | 2.3 | 4.7 | 2.7 | 4.0 | 2.0 | 4.3 | 1.7 | 4.3 | 2.3 | 4.0 |
| **11** | 2 | 2.0 | 2.7 | 2.3 | 3.0 | 2.0 | 3.0 | 2.3 | 2.7 | 2.0 | 2.7 | 2.0 | 2.3 |
| **12** | 2 | 2.0 | 2.3 | 2.0 | 2.0 | 2.0 | 2.7 | 2.3 | 2.0 | 2.3 | 2.0 | 2.3 | 1.7 |
| **13** | 2 | 2.3 | 2.0 | 2.0 | 2.0 | 2.0 | 2.0 | 1.7 | 1.3 | 1.7 | 2.3 | 2.7 | 1.7 |
